# Biomechanical response of decompression alone in lower grade lumbar degenerative spondylolisthesis--A finite element analysis

**DOI:** 10.1186/s13018-024-04681-4

**Published:** 2024-04-01

**Authors:** Renfeng Liu, Tao He, Xin Wu, Wei Tan, Zuyun Yan, Youwen Deng

**Affiliations:** Department of Spine Surgery, Central South University Third Xiangya Hospital, Changsha, China

**Keywords:** Degenerative lumbar spondylolisthesis, Laminectomy, Decompression alone, Biomechanics, Finite element analysis

## Abstract

**Background:**

Previous studies have demonstrated the clinical efficacy of decompression alone in lower-grade spondylolisthesis. A higher rate of surgical revision and a lower rate of back pain relief was also observed. However, there is a lack of relevant biomechanical evidence after decompression alone for lower-grade spondylolisthesis.

**Purpose:**

Evaluating the biomechanical characteristics of total laminectomy, hemilaminectomy, and facetectomy for lower-grade spondylolisthesis by analyzing the range of motion (ROM), intradiscal pressure (IDP), annulus fibrosus stress (AFS), facet joints contact force (FJCF), and isthmus stress (IS).

**Methods:**

Firstly, we utilized finite element tools to develop a normal lumbar model and subsequently constructed a spondylolisthesis model based on the normal model. We then performed total laminectomy, hemilaminectomy, and one-third facetectomy in the normal model and spondylolisthesis model, respectively. Finally, we analyzed parameters, such as ROM, IDP, AFS, FJCF, and IS, for all the models under the same concentrate force and moment.

**Results:**

The intact spondylolisthesis model showed a significant increase in the relative parameters, including ROM, AFS, FJCF, and IS, compared to the intact normal lumbar model. Hemilaminectomy and one-third facetectomy in both spondylolisthesis and normal lumbar models did not result in an obvious change in ROM, IDP, AFS, FJCF, and IS compared to the pre-operative state. Moreover, there was no significant difference in the degree of parameter changes between the spondylolisthesis and normal lumbar models after undergoing the same surgical procedures. However, total laminectomy significantly increased ROM, AFS, and IS and decreased the FJCF in both normal lumbar models and spondylolisthesis models.

**Conclusion:**

Hemilaminectomy and one-third facetectomy did not have a significant impact on the segment stability of lower-grade spondylolisthesis; however, patients with LDS undergoing hemilaminectomy and one-third facetectomy may experience higher isthmus stress on the surgical side during rotation. In addition, total laminectomy changes the biomechanics in both normal lumbar models and spondylolisthesis models.

## Introduction

Lumbar degenerative spondylolisthesis (LDS) is a common disorder of the spine, frequently observed in the elderly, which manifests as the upper vertebrae slipping relative to the lower vertebrae on the basis of images [1, 2]. The L3-4 and L4-5 segments are the most affected index segment [3]. In clinical scenarios, most of the patients suffer from lower back pain due to LDS; with the progress in slip, the condition is accompanied by pain and numbness in the lower limbs, leading to functional limitations [4]. Around 4.1% of individuals suffer from LDS globally [5], which lays a huge economic burden [6]. Surgical intervention is the traditional method used for patients who are not responding to conservative treatment options [6, 7].

Decompression combined with fusion, whether through open approaches or minimally invasive surgeries, has achieved excellent clinical results in patients with spondylolisthesis [8, 9]. Laminectomy combined with cage implantation assisted with screw-rod fixation can effectively release the pressure of the nerve root or dura well and also avoid the risk of iatrogenic instability [10]. However, concerns have been raised regarding the necessity of instrument fusion which can lead to additional costs, longer surgical time, more blood loss, and possible nerve root injury [11, 12]. Several studies have compared decompression alone with decompression united fusion and have shown that decompression alone can yield excellent clinical outcomes [1, 6, 12, 13]. However, some researchers believe certain patients with lower-grade LDS could benefit more from laminectomy combined with fusion, based on their studies [1]. The choice between the two procedures may depend on the patient’s symptoms and the segment stability, as decompression alone may not be suitable for mechanical back pain or unstable spondylolisthesis [3, 10]. Currently, there is contradictory evidence regarding the indications and clinical outcomes of the two surgeries based on published reports [12, 14]. In most cases, the choice between decompression alone or instrument fusion in treating LDS depends on the surgeon’s preference, as there is still insufficient evidence to evaluate which operative type is more effective [5]. While clinical studies comparing decompression alone with additional instrument fusion are common, there are very few studies comparing the biomechanics of the two surgeries.

The finite element method (FEM) is a powerful tool in the field of biomechanics and has been used to study spine biomechanics in the last decades [15]. FEM has several advantages when compared to in vitro experiments, including good repeatability and low cost. Additionally, it is easy for FEM to obtain the stress or pressure distribution of bone and soft tissue [15]. At present, the common biomechanical parameters include segmental ROM, IDP, AFS, FJCF, and IS. In this FE study, we evaluated the biomechanical characteristics of lower LDS after total laminectomy, hemi-laminectomy, and one-third facetectomy by analyzing the above parameters.

## Methods

### Intact L3-S1 finite element model

**Fig. 1:**
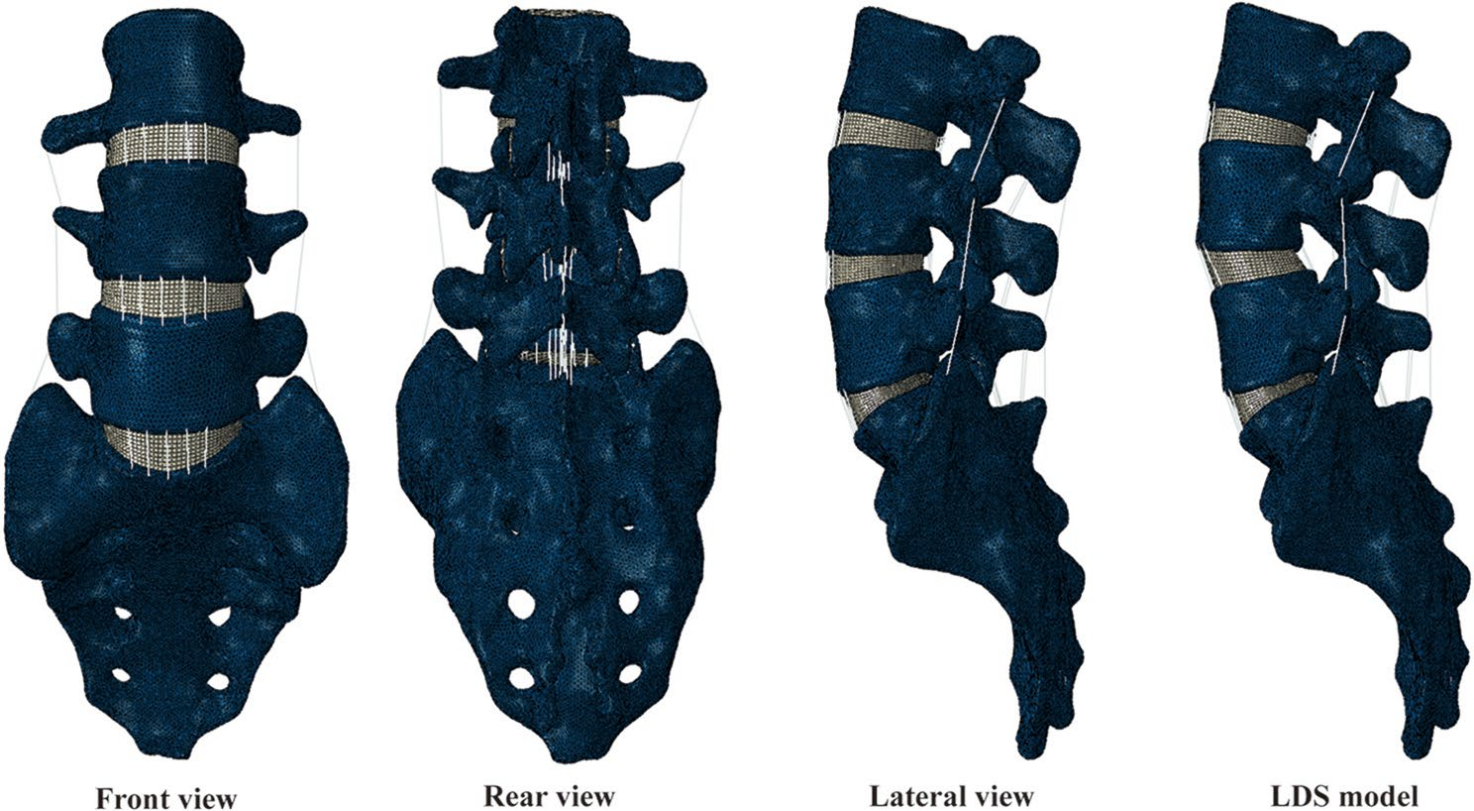
The normally intact L3-S1 model and lumbar spondylolisthesis model

**Fig. 2:**
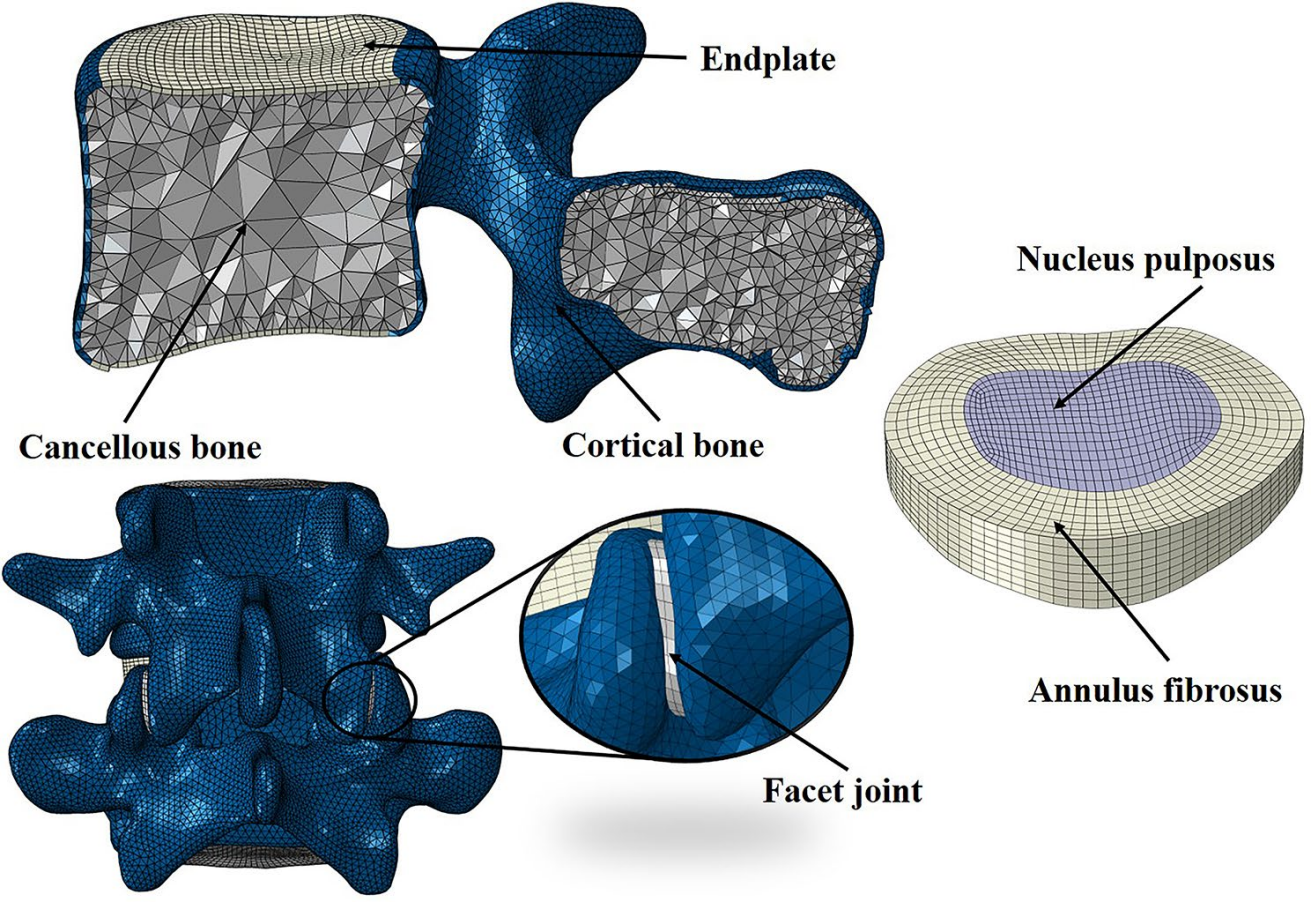
Details of the normally intact L3-S1 model

The L3-S1 finite element (FE) model was established based on high-resolution computed tomography images of a 27-year-old healthy male participant (height :175 cm, weight :70 kg). The L3-S1 geometric model was first constructed based on the lumbar CT data using the software Mimics 21.0 (Materialise Inc., Leuven, Belgium), Geomagic Wrap 2017 (Geomagic, Inc., Research Triangle Park, NC, United States), and SOLIDWORKS 2018 (Dassault Systèmes Inc., France). The intact FE model was then established by meshing the geometric model, checking the mesh quality, and FE preprocessing in the software of HyperMesh 2020 (Altair Engineering, Inc., Executive Park, CA, United States). The normally intact L3-S1 model is shown in Figs. 1 and 2, and the lumbar spondylolisthesis model is shown in Fig. 1.

The intact L3-S1 FE model included cortical bone, cancellous bone, cartilage, endplate, annulus fibrosus, nucleus pulposus, and seven major ligaments, namely, the anterior longitudinal (ALL), posterior longitudinal (PLL), ligament flavum (LF), supraspinous (SSL), interspinous (ISL), capsular (CL), and intertransverse ligaments (IL). The thickness of the endplate and cortical bone was set to 0.6 mm, and the cartilage material is elastic, with a joint gap of 0.5 mm. The major ligaments were assumed to be tension-only truss elements. The volume of the nucleus pulposus accounted for 40% of intervertebral disc [16]. The posterior cartilage was modeled as surface-to-surface friction contact with a friction coefficient of 0.1 [17]. A detailed description of the mechanical properties, the element type, and numbers is listed in Table 1.

**Table 1:** The mechanical properties in FE models [16–20]

Components	Element type	Young modulus [MPa]	Poisson ratio	Element numbers	Nodes Numbers/ Cross-sectional area [mm^2^]	Reference
Cortical bone	C3D4	E_xx_=11,300	V_xy_=0.484	172,529	57,992	[17, 18]
		E_yy_=11,300	V_yz_=0.203			
		E_zz_=22,000	V_xz_=0.203			
		G_xy_=3,800				
		G_yz_=5,400				
		G_xz_=5,400				
Cancellous bone	C3D4	E_xx_=140	V_xy_=0.45	205,959	53,421	[17, 18]
		E_yy_=140	V_yz_=0.315			
		E_zz_=200	V_xz_=0.315			
		G_xy_=48.3				
		G_yz_=48.3				
		G_xz_=48.3				
Sacrum	C3D4	5,000	0.2	232,755	58,374	[16]
Nucleus pulposus	C3D8H	1	0.4999	12,784	15,489	[18, 19]
Annulus fibrosus	C3D8H	4.2	0.45	13,440	17,280	[16, 18]
Endplate	C3D8R	100	0.4	7,717		[16, 20]
Cartilage	C3D8R	24	0.4	1,270	2,944	[16, 18]
ALL/PLL/IF/ISL/SSL/TL/CL	T3D2	20/5/5/1.5/5/10/7.5	0.3	20/20/20/6/3/3/24	63.7/7/14.1/14.1/10.5/0.6/10.5	[19, 20]

### Surgical FE models and spondylolisthesis model

**Fig. 3:**
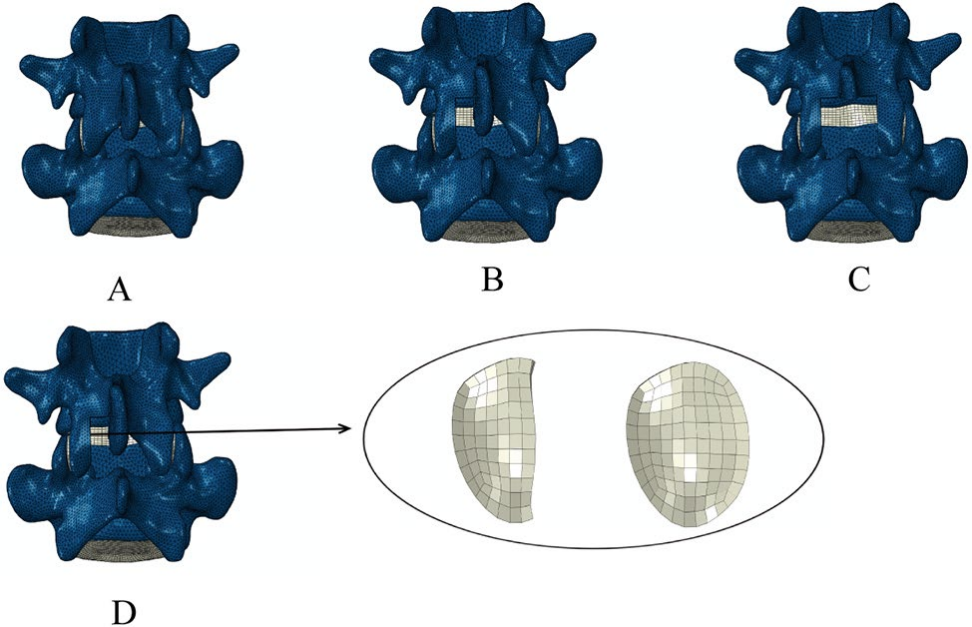
**A**, the intact spondylolisthesis model (Model B); **B**, the model of hemi-laminectomy; **C**, the model of total laminectomy; **D**, one-third facetectomy

Seven experiment models were constructed by modifying the intact FE model (model A). For the intact spondylolisthesis model (model B), L4 vertebrae slipped 5 mm to simulate grade I spondylolisthesis based on the Meyerding classification. The slipping part accounts for 1/7 of the length of the L5. To ensure the consistency of L4 vertebrae anteroposterior diameter, bilateral isthmuses were stretched in the spondylolisthesis models. In models A and B, a hemilaminectomy and total laminectomy were performed, respectively, at the L45 segment. In the hemilaminectomy models, the left side LF was removed; in the total laminectomy models, all the LF, SSL, and ISL were removed. The hemilaminectomy models were divided into models A1 and B1, and total laminectomy models were divided into models A2 and B2. For the 1/3 facetectomy, we only removed part of cartilage and CL, and didn’t remove any bony structure. The 1/3 facetectomy models were divided into models A3 and B3. All the modified models are shown in Fig. 3.

### Boundary and loading conditions

To simulated different types of movement, the normal mode wasl subjected to a 10 Nm applied to the L3 cranial endplate for flexion (FLE), extension (EXT), left lateral bending (LLB), right lateral bending (RLB), left axial rotation (LAR), and right axial rotation (RAR) movement. A vertical compression load of 400 N was applied to the central area of the L3 cranial endplate and kept vertical at all times [21]. During lumbar movement, the sacrum was fixed in all directions. The displacement of the intact model at 10 Nm was calculated, and the calculated displacement load was applied to the surgical models instead of the moment. All surgical models were analyzed using Abaqus 2020(Abaqus, Inc., Providence, RI, United States).

## Results

### Validation

**Fig. 4:**
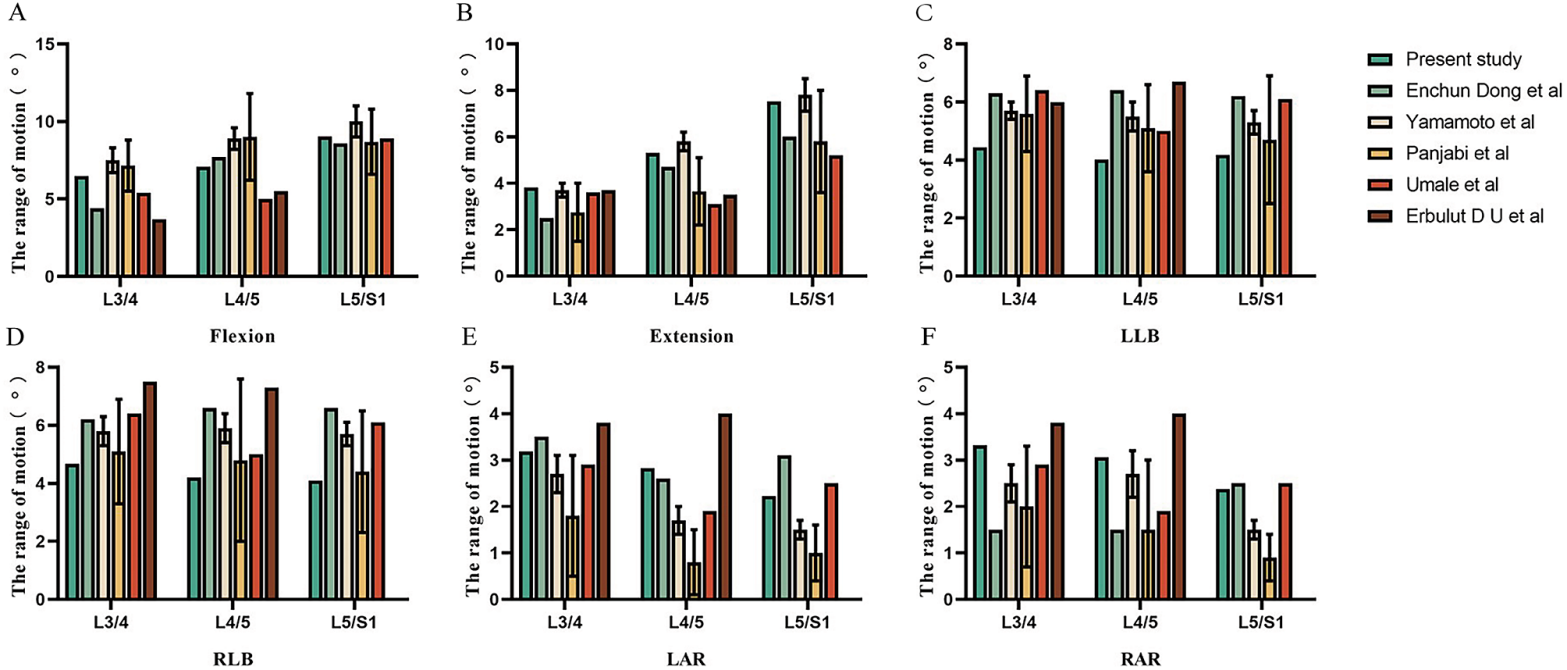
Comparison of ROM between the current models with previous studies. LLB, left lateral bending; RLB, right lateral bending; LAR, left axial rotation; RAR, right axial rotation

The loading condition for validation was the same as in previous studies (10 Nm pure moment). The ROM of each segment is illustrated in Fig. 4, and the predicted value of ROM was found to be consistent with previous results [22–26].

### ROM

**Fig. 5:**
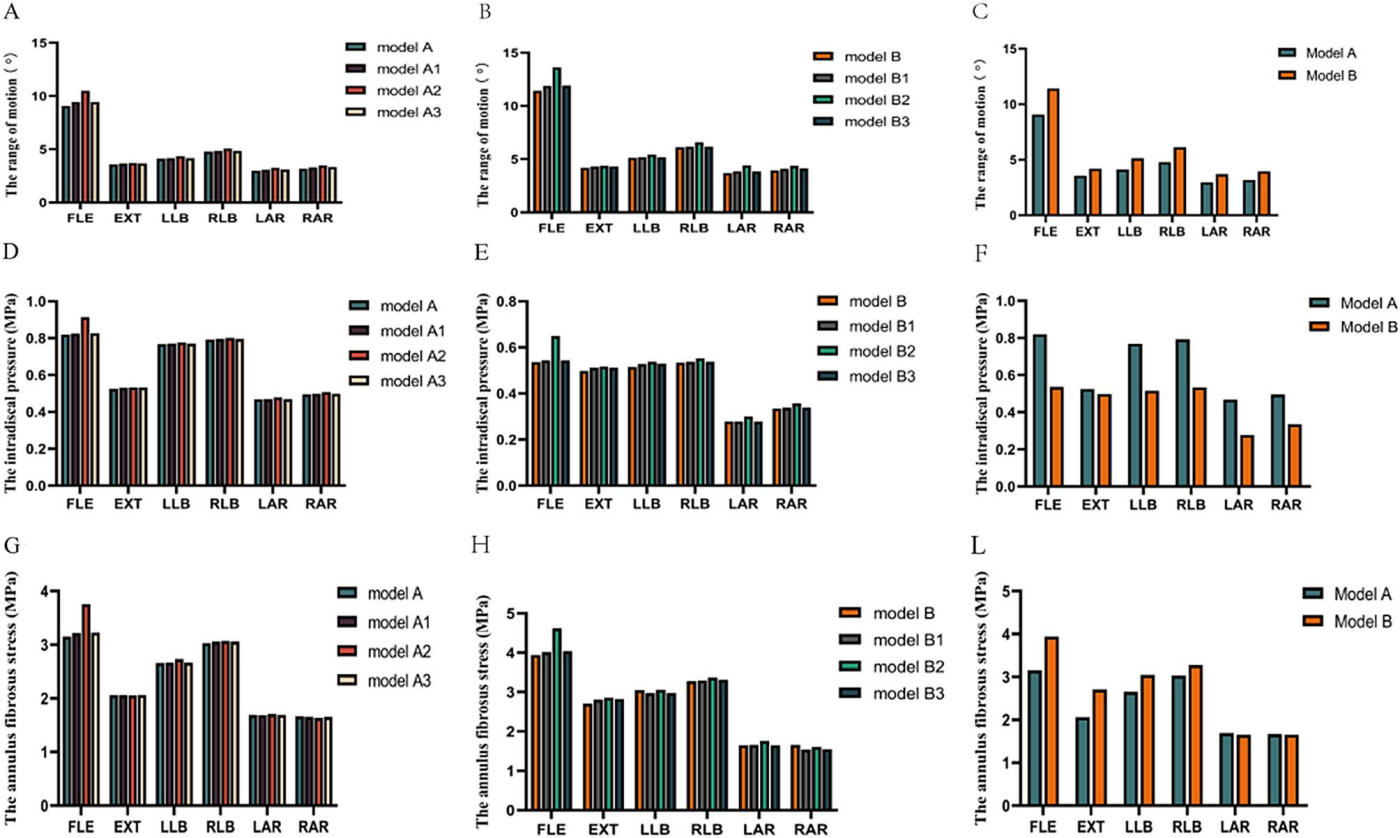
Comparison of ROM、IDP and AFS at the L4-L5 segment of different models. A-C indicates ROM, D-F indicates IDP, and G-L indicates AFS. The model A (intact normal model); the model A1 (hemi-laminectomy); the model A2 (total laminectomy); the model A3 (one-third laminectomy); The model B (intact spondylolisthesis model); the model B1 (hemi-laminectomy); the model B2 (total laminectomy); the model B3 (one-third laminectomy); FLE, flexion; EXT, extension

The A-C in Fig. 5 shows the different models of ROMs in the L4-L5 segments. The results indicated that hemilaminectomy and facetectomy had a relatively minor impact on segment stability, regardless of whether normal or spondylolisthesis models were used. In contrast, total laminectomy had a significant impact on stability. Additionally, for the same surgical condition, the movement level of spondylolisthesis models was slightly greater than that of normal lumbar models.

Compared to the intact normal model (model A), the ROM of the intact spondylolisthesis model (model B) increased by 21.69% (1.45°), 26.1% (1.67°), and 24.37% (0.53°) during flexion-extension, lateral bending, and rotation, respectively. In the lumbar normal models, the maximal motion of models A1 and A3 occurred during extension movement, with increasing rates of 3.71% and 4.17%, respectively. Compared to model A, the ROM of model A2 increased by 15.74%, 3.69%, 5.84%, 5.73%, 9.23%, and 9.37% during FLE, EXT, LLB, RLB, LAR, and RAR, respectively. Similarly, in the spondylolisthesis models, the maximal motion of models B1 and B3 occurred during extension movement, with the increase rates being 3.81% and 4.10%, respectively. Compared to model B, the ROM of model B2 increased by 19.2%, 3.68%,6.82%, 7.67%, 11.9%, and 10.58% during FLE, EXT, LLB, RLB, LAR, and RAR, respectively.

### IDP

The IDP changes in L4-L5 segments of normal lumbar spine model and lumbar spondylolisthesis model are shown in D-F of Fig. 5. According to the calculated results, no significant increase was observed in IDP after three decompression-alone procedures in all the models lateral bending and axial rotation. For models A1, model A3, model B1, and model B3, their maximal rate occurred in extension movement, the increase rates being 1.26%, 1.34%, 2.76%, and 2.79%, compared with models A and B, respectively. After total laminectomy, the IDP of model A2 increased by 11.44%, 1.49%, 1.11%, 1.23%, 2.53%, and 2.51% compared to model A during FLE, EXT, LLB, RLB, LAR, and RAR, respectively; while the IDP of model B2 increased by 20.98%, 3.83%, 4.56%, 3.41%, 8.09%, and 6.46% compared with model B during FLE, EXT, LLB, RLB, LAR, and RAR, respectively. However, there was a significant decrease in the tendency of the segmental IDP after the vertebrae slipped. The IDP of the model B decreased by 34.58%, 5.1%, 32.92%, 32.95%, 40.58%, and 32.35% during FLE, EXT, LLB, RLB, LAR, and RAR, respectively.

### Annulus fibrosus stress

**Fig. 6:**
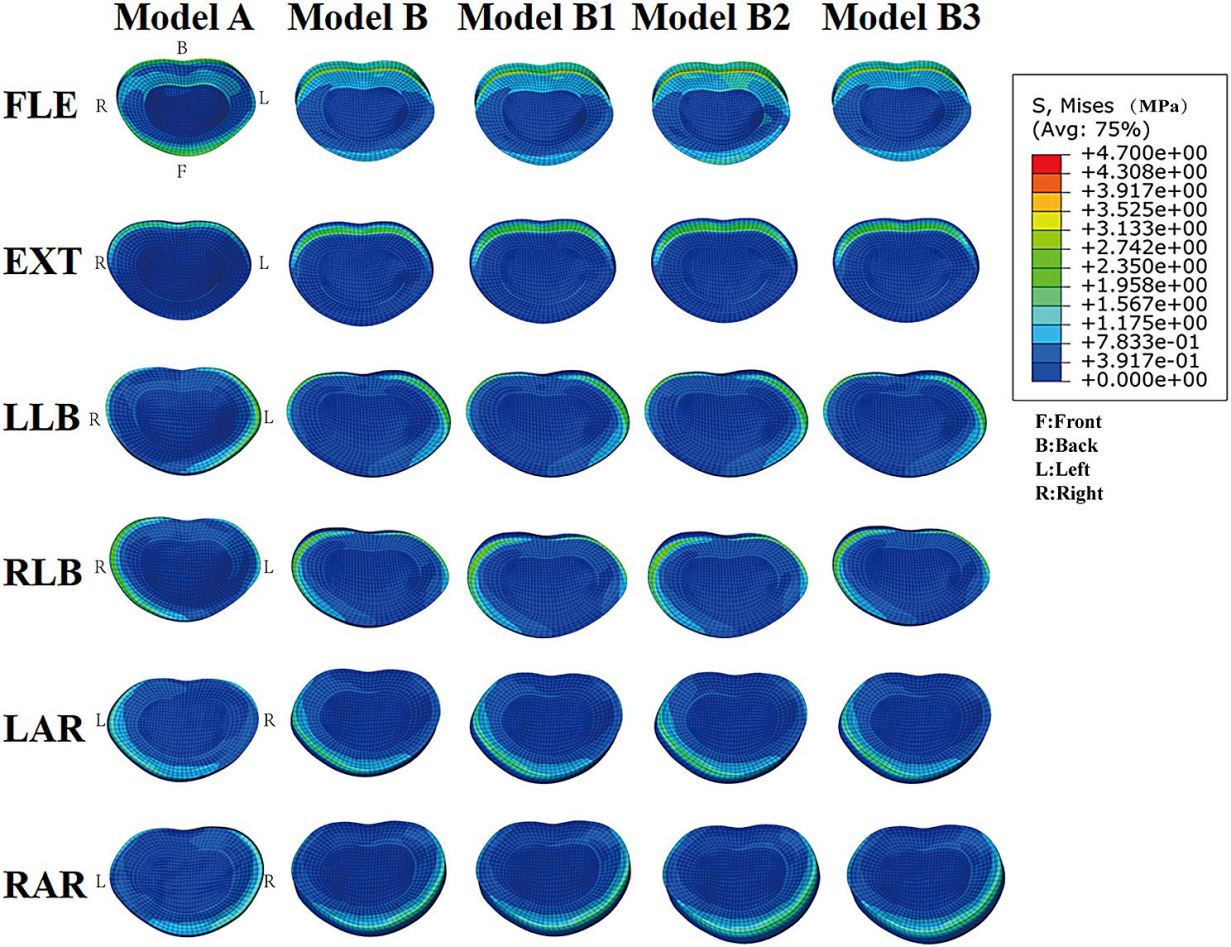
The stress is primarily distributed in the posterior-lateral region of the caudal intervertebral disc during flexion movement, and the stress is primarily distributed in the right anterior or left anterior region of the caudal intervertebral disc during axial rotation movement, while the stress is primarily distributed in the right posterior or left posterior region of the cephalic intervertebral disc during lateral bending movement

Compared with normal lumbar models, the annulus fibrosus stress (AFS) in the spondylolisthesis models presented a higher value for the same conditions. The AFS of model B increased by 25.02%, 31.54%, 14.64%, 8.11%, 13.60%, and 7.01% under FLE, EXT, LLB, RLB, LAR, and RAR, respectively, when compared to model A. In the normal lumbar models, the AFS of model A2 increased by 19.12% more than model A under flexion, with other motions having little influence on AFS. In the spondylolisthesis models, the AFS of model B2 increased by 17.14%, 5.5%, 2.75%, and 5.46% compared with model B under FLE, EXT, lateral bending, and axial rotation, respectively. The AFS comparison of different models at the L4-L5 segment is shown in G-L of Fig. 5, while the stress distribution of the disc at the L4-L5 segment is shown in Fig. 6.

### Facet joints contact force

**Fig. 7:**
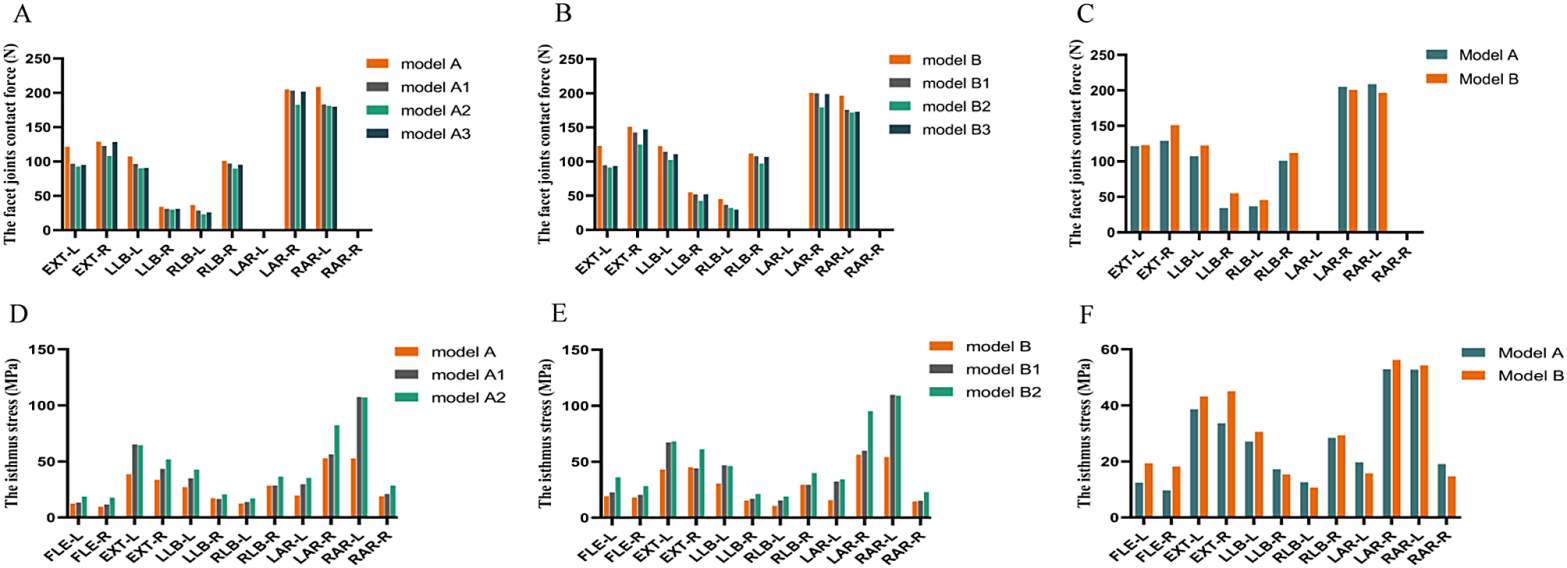
Figure A-C shows the FJCF, the “-L” presents the left facet joint, and “-R” presents the right facet joint. Figure D-F shows the IS, the “-L” presents the left isthmus, and “-R” presents the right isthmus

The comparison of facet joint contact forces of different models in segments L4-5 is shown in A-C of Fig. 7. The greatest facet joint contact force was observed during the axial rotation in all movements, followed by extension, and the FJCF of axial movement was above 200 N. For both normal lumbar and spondylolisthesis models, FJCF decreased after each of the decompression-alone procedures, with a more significant decrease observed with resection ranges. Except for rotation movement, the contact force of bilateral facet joints in model B showed an increase compared to model A; the value of FJCF in spondylolisthesis models was larger than that in normal lumbar models for the same surgical operation. In the normal lumbar models, the greatest decrease in FJCF occurred in total laminectomy, followed by facetectomy and hemilaminectomy; the same tendency also occurred in spondylolisthesis models. In addition, the greatest decrease degree of the FJCF occurred during extension (more than 20%), followed by lateral bending and rotation after the three surgeries in all surgical models. The decrease in FJCF after the same surgery was not significant between normal and spondylolisthesis models.

### Isthmus stress

The isthmus stress (IS) of different models at the L4-L5 segment is shown as D-F in Fig. 7. The results showed that the maximal stress occurred in rotational movement in all models, followed by extension, lateral bending, and flexion movements. There was an apparent increase in stress on the ipsilateral isthmus during lateral bending, while contralateral isthmus stress had a larger change under axial rotation.

**Fig. 8:**
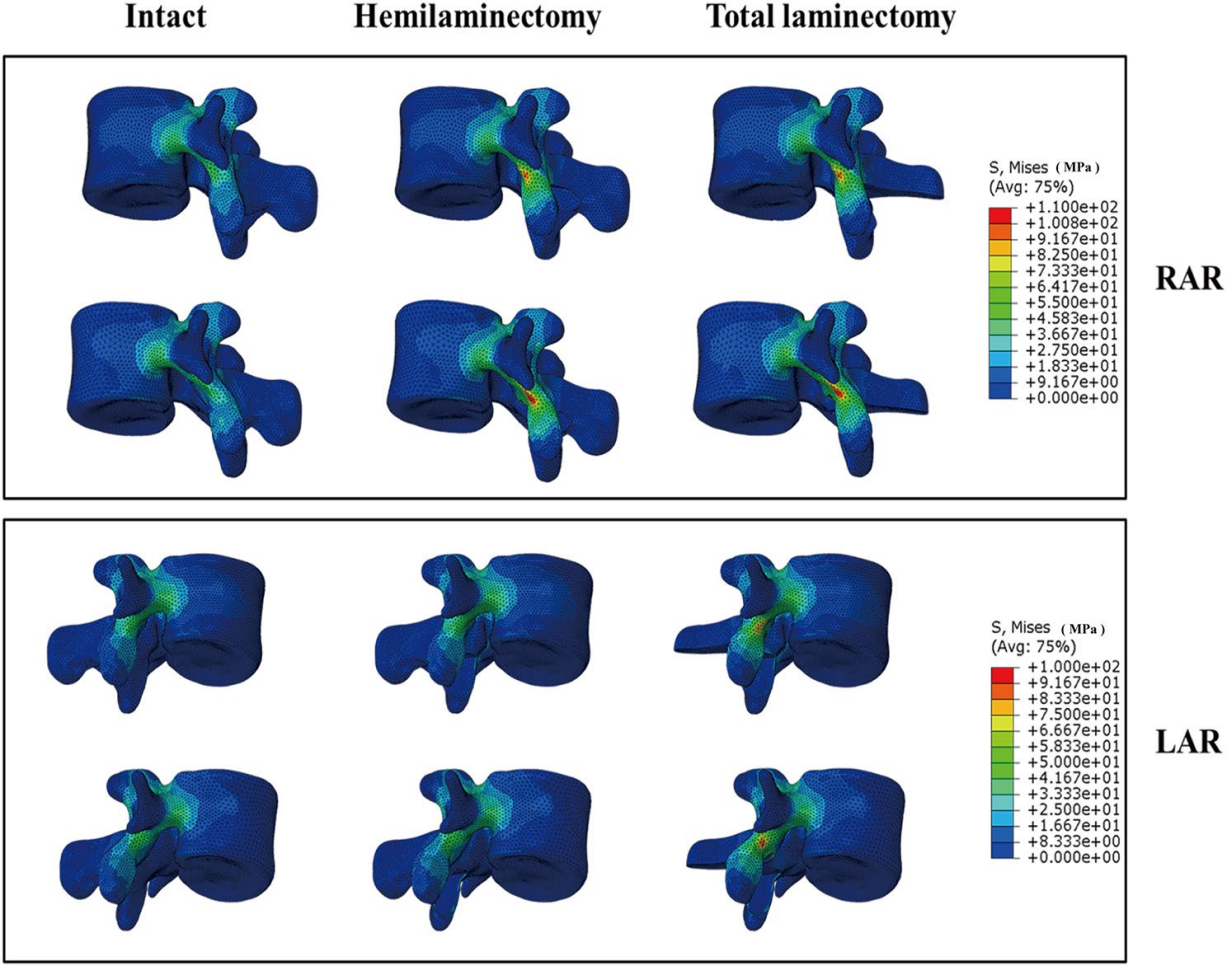
The stress distribution of isthmus during axial rotation. LAR, left axial rotation; RAR, right axial rotation

Compared to model A, the IS of model B increased at different levels, especially during flexion and extension movements, with average rates of 71.22% and 22.85%, respectively. For the hemilaminectomy, the stress of the resection side had an increase of more than 50% during extension and lateral bending in models A1 and B1, and the increasing rates were more than 110% during rotation. For the total laminectomy, the stress of the bilateral isthmus showed a huge increase in models A2 and B2 under all movements. Although the IS of spondylolisthesis models showed a larger value than normal lumbar models after hemilaminectomy and total laminectomy, there was no greater extent of increase to observe. The stress contour map of hemilaminectomy and total laminectomy under rotation is shown in Fig. 8.

## Discussion

In recent years, studies have shown that laminectomy alone also yields satisfactory clinical outcomes for LDS [12, 27], and some studies have reported that a higher rate of reoperation for laminectomy alone compared to laminectomy combined with instrument fusion in the postoperative [28]. However, there is a lack of biomechanical results of laminectomy alone for LDS. In this study, we developed a normal L3-S1 finite element model based on CT data and then constructed a lower-grade LDS model and several surgical models using simulation tools. The purpose of this study was to investigate the biomechanical characteristics of decompression alone for spondylolisthesis using parameters such as ROM, IDP, FJCF and IS calculated by FE software.

### The range of motion

Laminectomy is a common surgical method for lumbar stenosis. According to the results of finite element analysis and cadaveric specimen experiments [29, 30], unilateral laminectomy had a minimal impact on the segmental ROM. Zander et al. conducted finite element analysis by establishing a lumbar spine model to compare the biomechanical effects of graded facetectomy. They found that if facetectomy is performed in a graded manner, removing less than 50% of the bone, lumbar spine stability will not be significantly affected [21]. In the Burkhard et al. study, the segmental ROM after hemilaminectomy increased by 6% (5–10%), 3% (1–5%), and 12% (4–22%) during flexion-extension, lateral bending and rotation, respectively [31]. These studies collectively indicate that hemilaminectomy and facetectomy involving less than 50% of the facet joints have no apparent adverse effects on spinal stability, consistent with our findings. In our study, hemilaminectomy increased 3.18%, 1.15%, and 3.71% in flexion-extension, lateral bending, and axial rotation ROM, respectively. Hemilaminectomy and 1/3 facetectomy led to increases of 3.58%, 1.28%, and 4.17% in the mentioned ROM parameters. In addition, the ROM of the intact lower-grade spondylolisthesis model (model B) showed an obvious increase compared to model A, but hemilaminectomy or 1/3 facetectomy did not significantly change the ROM in the spondylolisthesis model. Considering that the anterior vertebral body bears a considerable portion of spinal stress, the buffering effect of intact intervertebral discs and the preservation of posterior midline structures such as facet joints and spinous processes compensate for removing partial bone structures and ligaments, we speculate that even with partial removal of bone structures and ligaments, spinal stability may not be significantly altered.

However, the index segment bears the risk of iatrogenic instability after total laminectomy if it lacks an additional fusion procedure. Postacchini et al. reported that 3 out of 32 patients suffered from significant segment disability after total laminectomy [32]. In Lener et al.‘s study [30], when complete laminectomy was performed with bilateral partial facetectomy, segmental ROM increased by 20% ± 15.9, 11% ± 9.9, and 19% ± 10.5% in flexion-extension, lateral bending, and axial rotation, respectively. In our study, due to the preservation of facet joints, the percentage increase in segmental ROM was smaller compared to cadaveric specimen experiments. After total laminectomy, segmental ROM increased by 9.71%, 5.79%, and 9.30% in flexion-extension, lateral bending, and axial rotation, respectively. These results suggest that in the lumbar spondylolisthesis model, the increase in ROM is greater compared to the normal lumbar spine model after total laminectomy, indicating that total laminectomy is not recommended in cases of lumbar spondylolisthesis. It is reported that the preservation of the dorsal midline structures could contribute to maintaining enough stability in the normal lumbar, bilateral laminotomy or unilateral laminectomy with “over the top” could be an alternative procedure when bilateral decompression is acquired [33–36]. In situations where total laminectomy is deemed necessary for decompression, it may be advisable to consider laminectomy with implantation techniques to reduce the risk of postoperative instability.

### Intradiscal pressure and annulus fibrosus stress

The intradiscal pressure embodies a response from the nucleus in a state of compression [37]. As the carrying load of the nucleus increases, the IDP also increases [38], indicating a higher possibility of nucleus degeneration [39]. The IDP did not show an obvious increase tendency in both normal lumbar and spondylolisthesis models after hemilaminectomy and 1/3 facetectomy. In contrast, the IDP of total laminectomy shows an obvious increase during flexion movement. In the normal models, the IDP increased by 0.09 MPa, and in the spondylolisthesis models, the IDP increased by 0.11 MPa. The above data show that total laminectomy could easily induce the degeneration of the nucleus pulposus compared with other procedures. Compared to the normal intact lumbar model, the IDP decreased significantly in the intact spondylolisthesis model. Disc degeneration is regarded as the inducement of segment stability loss and LDS [4]. The degenerative disc loses the ability to bind water under compression, which leads to a decrease in intradiscal pressure [40]. Because of the loss of intradiscal pressure, the annulus and nucleus will bear more shear stress, which could induce the annulus tear [40, 41].

Apart from carrying the load, the nucleus pulposus also induces tensile stress on the annulus fibrosus [37]. High stress may lead to a higher degeneration risk of annulus fibrosus; this study found that the AFS increased with the resection range. The stress of total laminectomy was higher level in both normal lumbar and spondylolisthesis models, which was consistent with previous studies [29]. The highest AFS occurred in model B2 during flexion, at 4.62 MPa, which is less than the failure strength of 8.5 MPa [15]. Although there was no significant increase in AFS compared to the intact spondylolisthesis model after facetectomy and hemilaminectomy in spondylolisthesis models, it is worth noting that the index segment AFS in intact spondylolisthesis model experienced an obvious rise compared to the intact normal lumbar model. Therefore, patients with spondylolisthesis may be at a higher risk of annulus degeneration.

### Face joints contact force

As a part of a three-joint complex, facet joints play a crucial role in maintaining spine stability, especially during extension and rotation movements [39, 42, 43]. Previous studies have shown that the FJCF is greatest during rotation, followed by extension and lateral bending, consistent with our findings [44, 45]. It is reported that the coronal angle of the facet joint gradually decreased and sagittal orientation increased with age, and the change of direction could lead to spondylolisthesis [46]. However, Leng et al. posit an interaction force between the lower vertebra’s superior articular process and the sliding vertebra’s inferior articular process, leading to the remodeling and morphological changes of the facet joints [47]. Morphological changes can weaken the resistance of the facet joints to anterior shear forces. When the forward shear force on the vertebra exceeds the resistance of the articular processes and posterior ligaments, it can result in lumbar spondylolisthesis. Changes in direction are a consequence of facet joint remodeling. In addition, the study of Liu et al. found The FJCF increased with the increase in the coronal angle of facet joints; they speculated that a bigger coronal angle of facet joints could contribute to bearing more mechanical load and maintaining spine stability [48].

In both normal lumbar and spondylolisthesis models, the greatest decrease in FJCF was observed with total laminectomy, followed by hemilaminectomy combined with 1/3 facetectomy and hemilaminectomy. The variation in FJCF was similar to the ROM. The capability of bearing load in facet joints is believed to be relevant to spine stability. However, high FJCF can induce facet joint arthrosis and painful articular facets [39, 49], as the normal facet joints can bear approximately 4-25% of the total load [49]. Park et al. found that a severe degenerative spine can cause a greater FJCF [50]. Similarly, the FJCF in the intact spondylolisthesis model was larger than the intact normal model in our results. We do not observe a significantly greater decrease in the FJCF in spondylolisthesis models compared to normal lumbar models under the same surgical condition. Therefore, we believe that stability loss in lower-grade LDS is acceptable after hemi-laminectomy and facetectomy.

### Isthmus stress

The isthmus was recognized as a weak area in the lumbar spine [37]. Spondylolysis is believed to result from repetitive mechanical stress on the lower lumbar vertebrae [51]. Excessive activity and stimulation of the fractured isthmus can lead to symptoms such as pain. While most individuals affected by these conditions are asymptomatic, a minority may experience chronic disabling lower back pain, sometimes radiating to the buttocks or thighs; this may be due to altered disc stress and increased disc degeneration following isthmic fracture, leading to chronic irritation [52, 53]. Despite most surgical interventions targeting the involved motion segment, some patients may continue to experience or exacerbate symptoms even after successful bony fusion of the affected segment. Studies have shown that partial isthmic resection may increase pressure in the area [45], increasing the risk of isthmus fracture. In a study by Spina et al., it was found that more than 75% of the isthmus resection would cause the IS to approach the ultimate strength (120–140 MPa) of cortical bone; they suggested that surgeons should avoid resecting more than 50% of the isthmus [45]. We performed a pure laminectomy without destroying the isthmus, which is similar to the 0% isthmus resection in the Nicholas et al. study [45]. Our results showed that the maximal stress in the isthmus was 109.80 MPa during rotation, which is lower than the ultimate strength. However, excessive rotation moments should still be avoided. Overall, the isthmus exhibited higher stress in spondylolisthesis models and may have a higher risk of isthmus fracture during vigorous exercise.

### Limitations

Some limitations in our study should be acknowledged. First, there is no suitable method of validation for developing a spondylolisthesis model, so we developed our spondylolisthesis model based on normal lumbar spine by extending the isthmus, without considering the issue of ligament pre-tension, which may not accurately reflect the morphological characteristics of lower-grader LDS. It is reported that the tropism and morphology of the facet joint could change in the LDS, which could influence the biomechanics of the motion segment [47, 54]. Therefore, it may not fully simulate the true physiological status of spondylolisthesis. Second, due to the complexity in vivo, we simplified the model in the process. Therefore, the FE results should be considered to have a similar tendency to the actual situation and provide a possible consequence in clinical settings but not present the same mechanical behavior as in vivo. The FE results should be considered to have a similar tendency to the actual situation and provide a possible consequence in clinical settings but not present the same mechanical behavior as in vivo. Besides, there may be individual differences in each lumbar CT scan. Including differences in the height of disc space, the facet joint tropism, and bilateral asymmetry of the vertebral body, which could lead to diverse outcomes. Thus, developing multiple finite element models by adding CT data could increase the credibility of the results. Additional samples or in vitro experiments are needed to validate our findings in the future.

## Conclusion

This study suggests that hemilaminectomy and one-third facetectomy may be viable surgical options for lower-grade LDS, with minimal impact on segment stability. However, patients with LDS undergoing hemilaminectomy and facetectomy may experience higher isthmus stress on the surgical side during rotation. In addition, total laminectomy changes the biomechanics of both normal lumbar and spondylolisthesis models.
